# Determination of Formaldehyde by HPLC with Stable Precolumn Derivatization in Egyptian Dairy Products

**DOI:** 10.1155/2018/2757941

**Published:** 2018-11-08

**Authors:** Ahmed Salem Sebaei, Ahmed M. Gomaa, A. A. El-Zwahry, E. A. Emara

**Affiliations:** ^1^QCAP Laboratory, Agricultural Research Center, Ministry of Agriculture, Giza, 12311, Egypt; ^2^Dairy Technology Department, Animal Production Research Institute, Agricultural Research Center, Ministry of Agriculture, Giza, 12311, Egypt

## Abstract

Formaldehyde is one of the most dangerous chemical compounds affecting the human health; exposure to it from food may occur naturally or by intentional addition. In this study a high performance liquid chromatography method for determination of formaldehyde in dairy products was described. The dairy samples were reacted and extracted with a warmed organic solvent in the presence of derivatizing agent 2,4-dinitrophenylhydrazine (DNPH) and formaldehyde; the mixture was centrifuged and followed by diode array detection. The method is validated and gives average recovery of formaldehyde at the three different levels 0.1, 5.0, and 10.0 mg/kg varied between 89% and 96%. The method is linear from the limit of quantification 0.1 mg/kg up to 10 mg/kg levels. This method is intended for formaldehyde analyses in dairy products simply with stable derivatization, minimum residue loss, excellent recovery, and accurate results with a sensitive limit of detection 0.01 mg/kg. 90 dairy samples from milk, cheese, and yogurt were investigated from seven Egyptian governorates and all samples were free from formaldehyde.

## 1. Introduction

Formaldehyde is an environmentally widely chemical compound that is carcinogenic to humans [1]. Exposure to formaldehyde may cause adverse health effects. It is the most observed contact allergen in metal working fluids [2]; toxic incident can cause environmental hypersensitivity and chronic degenerative disease [3]. Formaldehyde travels in the blood throughout the body and reacts with proteins, destroying their biological function. Also it can react with an amine functional group of the amino acid lysine in a protein, called rhodopsin. Formaldehyde also reacts with amino groups in other proteins, including many enzymes, and the loss of the function of these biological catalysts causes death [4].

Formaldehyde is used for preparation of the hardest common plastics such as electric insulators, decorative laminates, tableware, and glass fiber [5]. It has been frequently used to disinfect laboratories and hospital rooms and surgical instruments and as a preservative in medical laboratories and is widely used for the manufacture of building materials, adhesives for wood products, glues, paints and coatings, paper products, nail care and hair smoothing products, textiles and resins such as urea-formaldehyde, synthetic polymers, fertilizers, and pesticides [6].

**Figure 1:**
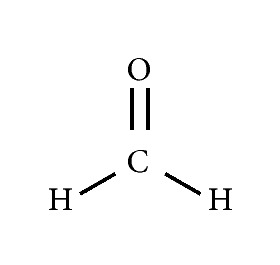
Chemical structural of formaldehyde.

Formaldehyde is a colorless, flammable gas and becomes liquid at room temperature and has a strong smelly unique odor. It is the simplest compound with carbonyl group as in Figure 1, CH_2_O. It is freely soluble and stable in water; the proton of the water bonds to the oxygen of the carbonyl group; the hydroxide ion combines to the carbon atom. Formaldehyde is over 99% hydrated. The hydrate of formaldehyde, called formalin, was once used to preserve biological specimens but it is no longer used because of carcinogenetic action [4].

Humans are exposed to formaldehyde by breathing, by ingestion, and dermally which has been confirmed by a variety of toxicity and monitoring studies [7, 8]. It can be found indoors and outside in naturally occurring and man-made materials. It is a by-product of the combustion process and the contact can be with cigarette smoke, home and office products, utensils products, cosmetics, and food. Dermal contact can result in irritation of the skin, eyes, nose, and throat [9].

While exposure to high levels of formaldehyde is dangerous, the concern is mostly at the occupational level where the EPA has set strict standards [10] on how much workers can be exposed to it in a given day. The European Union also is setting a maximum level in a directive (EU directive 95/2/EC) for residual of formaldehyde as hexamethylenetetramine in cheese at 25 *μ*g/kg and in another directive (EU directive 2009/10/EC) for the residual in alginate salts at 50 *μ*g/kg.

Several analytical techniques were proposed for formaldehyde determination in various food commodities and water including HPLC [11–13], GC [14, 15], TLC [16, 17], spectrophotometric [18, 19], and other colorimetric and electrochemical techniques [20–23]. The current work is intended to develop, validate, and apply a sensitive test method to determine the free formaldehyde by chemical derivatization with 2,4-dinitrophenylhydrazine (DNPH) in dairy products marketed in the biggest Egyptian governorates.

## 2. Materials and Methods

### 2.1. Chemicals and Materials

All chemicals and reagents were of HPLC or analytical grade. Deionized water used throughout the determinations was obtained from Milli-Q A10. Methanol and acetonitrile were with assay > 99%. Standard of formaldehyde (36% methanol stabilized solution) and 2,4-dinitrophenylhydrazine (99%) were purchased form Sigma Aldrich. All performance parameters and statistical experiments were applied on marketed processed cheese samples.

### 2.2. Samples Collection

90 dairy food samples were randomly collected from seven Egyptian governorates: Giza, Cairo, Sharqia, Damietta, New Valley, Beni Suef, and Gharbia. Samples were varied between UHT milk, plain milk, yogurt, and cheese and purchased from retail sources or markets. Samples were collected in the period from January 2016 to July 2017.

### 2.3. Standard Derivatization and Calibration Preparation

Two grams of the derivatizing agent DNPH was dissolved in 1 liter of acetonitrile: methanol 50:50; the pH of the mixture was adjusted by 10% phosphoric acid to the range of 5-6. This solution was used for formaldehyde standards derivatization and in sample preparation. Calibration levels 0.1, 0.5, 1.0, 5.0, and 10.0 mg/L were prepared in HPLC vials containing 100 *μ*L DNPH by suitable diluting of the working standard 100 mg/L in acetonitrile: water (50:50 v/v) and were kept stable for seven days; 25 *μ*L of the solution was subjected to HPLC analysis and the correlation coefficient had to be greater than 0.99.

### 2.4. Sample Preparation

10 grams of well homogenized milk or milk product was added to a 50 mL plastic bottle and 10 mL DNPH solution was added to the plastic bottle and the mixture was warmed to 70°C for 30 min in a shaking water bath and centrifuged at 4000 rpm for 10 min. The extraction reaction of formaldehyde was performed during the shaking in the heated water bath and after the centrifuging the mixture broke apart into two phases: the lower is solid and the upper is the extract aliquot. After filtration of the aliquot supernatant over membrane filter with pore size 0.45 *μ*m, 25 *μ*L of it was injected onto HPLC.

### 2.5. HPLC Analysis

The high performance liquid chromatography instrument used was model HP Agilent 1200 series from Germany equipped with a quaternary pump (G1311A), vacuum degasser (G1379B), autosampler (G1313A), fluorescence detector Agilent 1260 infinity/1200 series (G1321A), and analytical column: Agilent Eclipse plus C18 5 um 4.6× 250 mm. The software used was Chemistation for LC, Rev. B. 04.03 [20]. The HPLC-pump flow rate was 0.8 mL/min. Formaldehyde mobile phase was acetonitrile 50: water 50 (v/v). Detector parameters were diode array detector at 355 nm wavelength.

## 3. Results and Discussion

### 3.1. Optimization of HPLC Analysis

The described test method was developed and optimized for all procedure steps with some statistical justifications that enhance and optimize the method recovery, minimize time and reagents, and reduce matrix interference appropriately. The HPLC working wavelength of diode array detection was selected carefully after general scanning of formaldehyde derivatized standard from 150 to 900 nm and the maximum absorbance was given at 355 nm. Various mixtures of mobile phases were utilized for superior separation and the most preferable mixture was methanol: acetonitrile (50: 50 v/v) which gives the maximum performance parameters from intensity, resolution factor > 2.0, symmetry > 0.90, and run time (18 min).

### 3.2. Derivatization

**Figure 2:**
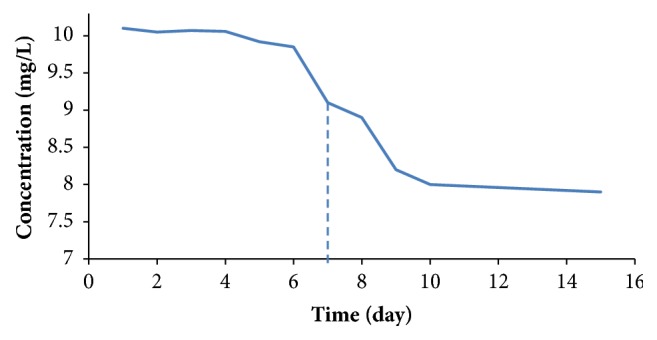
Stability of formaldehyde derivatized product over 2 weeks for 10.0 mg L formaldehyde standard.

DNPH has been used for its effectiveness in the interaction with the formaldehyde and produces a stable compound; it was prepared to be in the extraction solvent which enhances with methanol the precipitation of protein and fat contents in dairy samples. For pH adjustment, the four acids hydrochloric, sulfuric, acetic, and phosphoric were utilized; phosphoric acid gives the best results while sulfuric and hydrochloric acids gain more sample matrix which appears in the same retention time of formaldehyde peak; the acetic acid is not used because it stimulates the reaction slowly, leading to increased extraction time more than 1 hour. Phosphoric acid with 10% concentration attains the derivatizing agent extract solvent pH range of 5-6 and simulates the extraction reaction for formaldehyde faster than the other acids. The stability of the formaldehyde derivatized product was assessed by checking the amount of the peak of derivatized 10.0 mg/L standard and Figure 2 shows that the formaldehyde derivative product stable for 1 week giving 10.0 ± 1.0 mg/L accepted intermediate HPLC check over the first seven days under room temperature.

### 3.3. Effect of Reaction Temperature and Time

**Figure 3:**
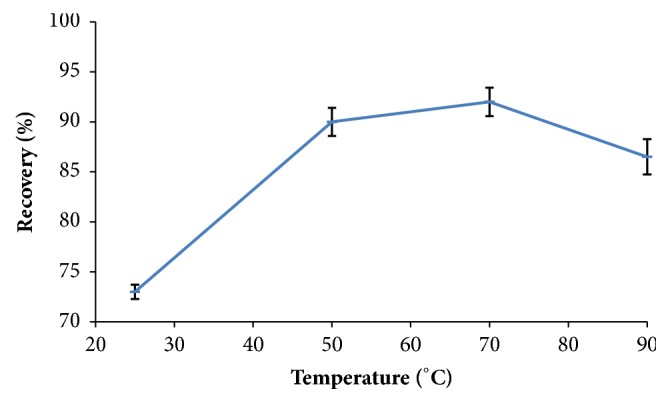
Effect of various reaction temperatures on formaldehyde recovery (mean ± sd, n = 2).

**Figure 4:**
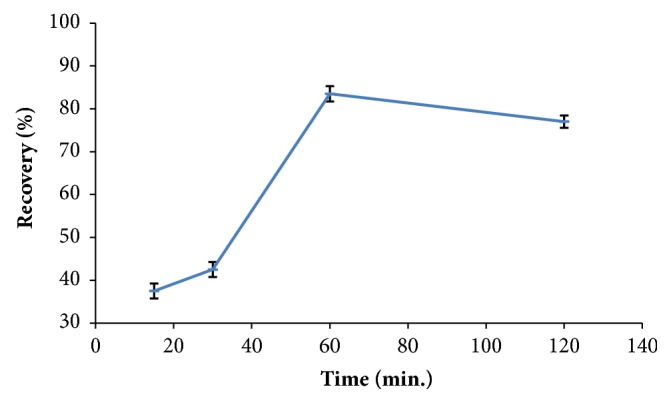
Effect of various times of formaldehyde incubation reaction recovery (mean ± sd, n = 2).

In order to optimize the excellent extraction conditions the effects of temperature and time were assessed for the highest recovery which was expressed by mean recovery from two replicates for each experiment result.  Figure 3 exhibits that the extraction of formaldehyde from dairy food increased generally as the extraction temperature increased (while the time was fixed to 60 min for the four experiments) till it reached the maximum recovery at 70°C and the recovery decreased significantly at 80°C.  Figure 4 exhibits a significant recovery trend relative to the time of extraction (while the temperature was fixed at 70°C). Recovery reached a maximum value at 60 min and decreased significantly at the incubation time 120 min. This study suggested that an efficient extraction could be with the conditions of 60°C and 60 min for excellent reaction recovery.

### 3.4. Method Validation (Fit for Intended Use)

This includes all of the parameters that demonstrate that a particular method used for quantitative measurement of analytes is reliable and reproducible for the intended use. EURACHEM [24] guideline was followed for checking the method validation performance parameters summarized in Table 1.

#### 3.4.1. LOQ and LOD

Limit of quantification (LOQ) is the lowest level of analyte that can be determined with acceptable performance. Acceptable performance is variously considered by different guidelines to include precision, precision and trueness, or measurement uncertainty. The accuracy of formaldehyde peak (response) was identifiable, discrete, and reproducible with a precision of 7%. Limit of detection (LOD) is the minimum concentration of analyte that can be detected with acceptable certainty, the level at which detection of the analyte becomes problematic. For this purpose the “3s” of the lowest quantifying level approach shown in Table 1 is the test method LOD. The method LOQ (0.1 mg/kg) was represented practically at lower than European Union maximum permitted limits (EU directive 95/2/EC and EU directive 2009/10/EC) for formaldehyde in cheese and it is worth mentioning that there is no regulation for formaldehyde in dairy products in Egypt. The developed method was sensitive, with a detection limit 0.01 mg/kg more sensitive than that reported by Wahed P. et al. [11] and comparable with Kaminski J. et al. [25].

#### 3.4.2. Precision and Trueness

Measurement “trueness” is an expression of how close the mean of an infinite number of results (produced by the method) is to a reference value. Since it is not possible to take an infinite number of measurements, trueness cannot be measured. We can, however, make a practical assessment of the trueness. This assessment is normally expressed quantitatively in terms of bias. The method trueness was checked by old proficiency test material with a known accepted concentration range. The proficiency test for 3% aqueous acetic acid sample was provided from FAPAS instead of dairy food. The aqueous acetic acid sample was treated by the same described test method and gives excellent accepted result. Because of unavailability of dairy products proficiency test sample with formaldehyde statistical trueness calculation can also be estimated by spiked samples at different levels on commercial dairy sample and bias expressed as absolute relative difference percent (RD %) must not exceed 20% (Table 1). Precision is the degree of agreement of replicate measurements under specified conditions. The precision is described by statistical methods such as standard deviation or confidence limit and less precision is reflected by a larger standard deviation and classified as repeatability and reproducibility shown to be 3 and 6% in milk, 4 and 8% in cheese, and 6 and 9% in yogurt, respectively, as in Table 2, less than 10% in agreement with [24].

#### 3.4.3. Method Linearity and Test Recovery

Method linearity was checked by making recovery tests at three different levels of 0.1, 5.0, and 10.0 mg/kg on cheese. The method was found to be linear from the limit of quantitation, 0.1, up to 10 mg/kg with a strong correlation coefficient 0.9998. The check for method linearity was performed with test recoveries for six replicates at the three different levels on dairy food samples. As reported in Table 1, the method has an excellent recovery varied between 89% and 96% over the three matrices which is also in agreement with [24] recommendations between 80 and 120% and higher than that reported by Kaminski J. et al. [25] who used the same presented extraction reaction followed by HPLC detection in milk.

#### 3.4.4. Measurement Uncertainty

The parameter associated with the result of a measurement that characterizes the dispersion of the values that could reasonably be attributed to the measured value. The measurement uncertainty seeks to investigate the major accuracy of the test method. Uncertainty was estimated (at the 95% confidence level and coverage factor of k = 2) to be in the range of ±24 to ±26% (Table 2) over the three dairy products matrices which are lower than that recently reported by Wahed P. et al. [11] who also describe the same presented reaction for extraction and liquid chromatographic detection in food. Bias reported from uncertainty using t-test statistical calculations shows that the method recovery is significantly different from 100% so the analytical result must be reportedly corrected for recovery for controlling compliance according to [26].

### 3.5. Formaldehyde Incidence in Dairy Products

Through the conducted approach, 90 samples of dairy products were tested for the presence of formaldehyde by the developed method. The 90 samples were clear from any trace of formaldehyde. The absence of any amount of detected formaldehyde is an advantage that eases off the risk of exposure from dairy products.

## 4. Conclusion

A reliable and accurate test method was presented here for formaldehyde monitoring in dairy products. The main insisting reason for this test method is the serious toxicological effects of formaldehyde on humans from food. This study aimed to optimize HPLC method without interference, with minimum reagent, and with fastness and reliability for routine analyses. The method was developed to extract formaldehyde perfectly from dairy samples by DNPH with optimized reaction conditions of 60 min at 60°C. The stability of formaldehyde derivative is tested and it can reasonably remain stable for one weak. Certain method validation parameters were assessed to investigate the method performance. The results of validation characteristics were excellent and confirm that the method is fit for the purpose. The developed method permits the detection of formaldehyde residues at 0.01 mg/kg. The method is recommended for use in routine determination of formaldehyde residue in dairy food.

## Figures and Tables

**Table 1:** Validation parameters for formaldehyde in dairy food.

Contaminant	Commodity	Spiking level (mg/kg)	Recovery (%)	Bias (R.D. %)^1^	LOD (mg/kg)	LOQ (mg/kg)	FAPAS®PT^2^
							1271
							Accepted Range
							(10.7–16.6) mg/kg
Formaldehyde	Cheese	0.1	95.0 ± 7.1	5.0	0.02	0.1	13.0
		5.0	91.7 ± 6.8	8.3			
		10.0	89.1 ± 6.1	10.9			
	Milk	0.1	95.3 ± 6.6	4.7	0.01	0.1	
		5.0	92.3 ± 6.5	7.7			
		10.0	90.2 ± 6.0	9.8			
	Yogurt	0.1	94.0 ± 7.7	6.0	0.02	0.1	
		5.0	92.8 ± 6.9	7.2			
		10.0	89.5 ± 6.7	10.5			

1: Relative difference.

2: Proficiency test.

**Table 2:** Precision contribution to the measurement uncertainty.

Contaminant	Commodity	Repeatability (%), RSD*∗*	Reproducibility (%), RSD#	Measurement uncertainty (%)
Formaldehyde	Cheese	4	8	± 25
	Milk	3	6	± 24
	Yogurt	6	9	± 26

*∗*: Relative standard deviation of repeatability was performed with n=6 by the same the personnel at the same time.

#: Relative standard deviation of reproducibility was performed with n=10 by different personnel at different times.

## Data Availability

The data used to support the findings of this study are available from the corresponding author upon request.
